# Cowden syndrome. Managing multiple skeletal metastases of different origin: a case report

**DOI:** 10.1186/1757-1626-1-265

**Published:** 2008-10-23

**Authors:** Antonios Angoules, Evangelia Maria Tsapakis, Ioannis Polyzois, Zakareya Gamie, James Julian Rankine, Eleftherios Tsiridis

**Affiliations:** 1 Academic Orthopaedic Unit, Leeds General Infirmary, Leeds School of Medicine, Leeds, LS1 3EX, UK; 2 Institute of Psychiatry, King's College London, London, UK

## Abstract

**Background:**

Cowden Syndrome is a rare autosomal dominant multiple hamartomatous condition, characterised by both benign and malignant tumours affecting multiple systems.

**Case presentation:**

We present a 47-year-old female patient with thigh pain that was diagnosed with Cowden syndrome 20 years ago and developed multiple and different skeletal metastases which became resistant to radio-chemotherapy. A percutaneous plate fixation of the distal femur with an intralesional excision and cementoplasty of the metastasis was performed initially. This was combined with a cemented total hip arthroplasty using an Exeter long revision stem and a cementoplasty of the femoral canal for the proximal lesions.

**Conclusion:**

A meticulous approach to her complex metastatic disease resulted in successful palliative prophylactic reconstructive surgery that improved her quality of life, allowing her independent pain free walking for 12 months.

## Background

Cowden Syndrome (CS) is a rare autosomal dominant disorder characterised by multiple hamartomas and an increased risk of breast, thyroid and endometrial carcinomas [1,2] Germline mutations in the tumour suppressor gene *PTEN *(phosphatase and tensin homolog) chromosome 10q23.2, which codes for a lipid phosphate mediating cell cycle arrest and apoptosis, were first described in Cowden Syndrome[3] In the next years since *PTEN *had been identified as the major CS susceptibility gene[4], a number of inherited cancer and developmental syndromes were associated with germline *PTEN *mutations. It has therefore been proposed that all such syndromes based on molecular defects are classified as *PTEN *Hamartoma Tumour Syndromes (PHTS)[2,5]. Irrespective of clinical phenotype however, the discovery of a germline *PTEN *mutation should trigger cancer risk surveillance[6]. Skeletal metastases of multiple origins are difficult to treat and palliative surgery usually requires a combination of approaches and techniques.

## Case presentation

A 47-year-old female Caucasian patient presented with a right sided limp and ipsilateral thigh pain with an established diagnosis of CS according to the International Cowden Syndrome Consortium Operational Criteria1, positive for tumour suppressor phosphatase and tensin homolog gene (*PTEN*) mutation.

She was first diagnosed with thyroid follicular carcinoma and was treated with a thyroidectomy in 1989. The patient then developed breast cancer in 1991 for which she underwent lumpectomy followed by adjuvant radio-chemotherapy. Due to local recurrence the following year, she was treated with a left mastectomy and a few months later she underwent right nephrectomy for renal carcinoma. In 2003, she was diagnosed with left scapular renal metastatic and during the same year she developed brain metastases that required a right frontal lobe resection followed by whole brain radiotherapy. On presentation, she was known to have pulmonary and multiple bone metastases. Palliative skeletal radiotherapy was completed having exceeded the maximum radiation limits. As a last resort, she was on interferon treatment as well as bisphosphonates for palliation therapy.

**Figure 1:**
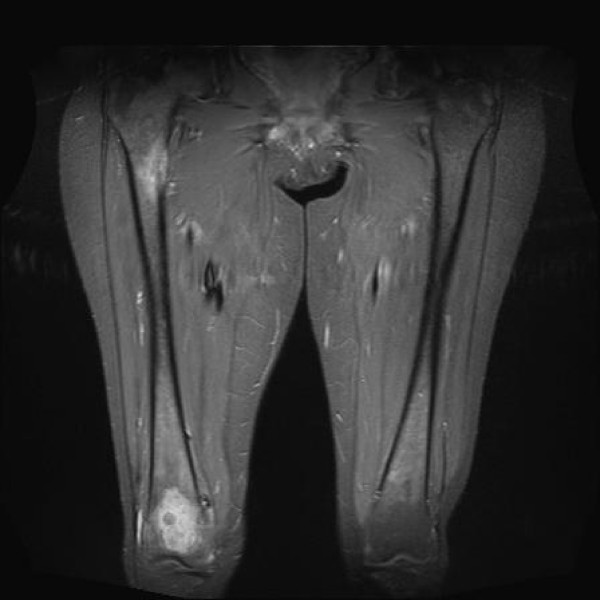
**T1 weighted fat suppressed post gadolinium coronal views of the whole right femur.** Vascular lesions are shown at the distal intercondylar femoral site and the proximal femur adjacent to the lesser trochanter.

**Figure 2:**
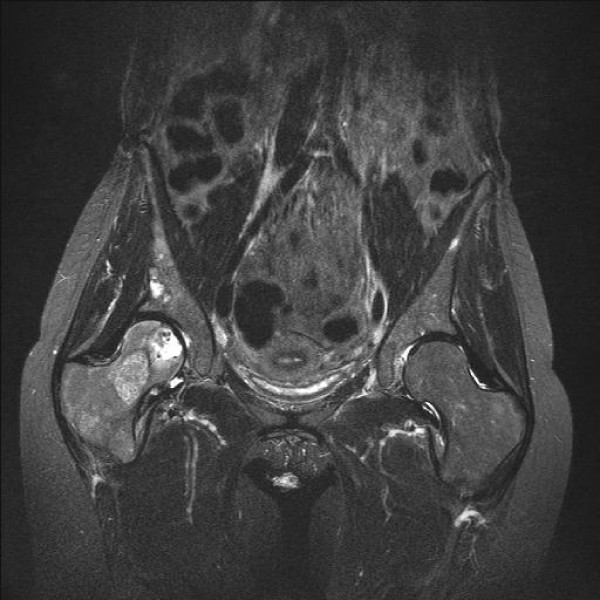
**Coronal T2 weighted fat suppressed (STIR) view of the right hip joint.** Two different signals in the femoral neck. The most proximal was similar to subtrochanteric and distal femoral intercondylar signal most possibly of vascular origin. The most distal lesion was less vascular.

**Figure 3:**
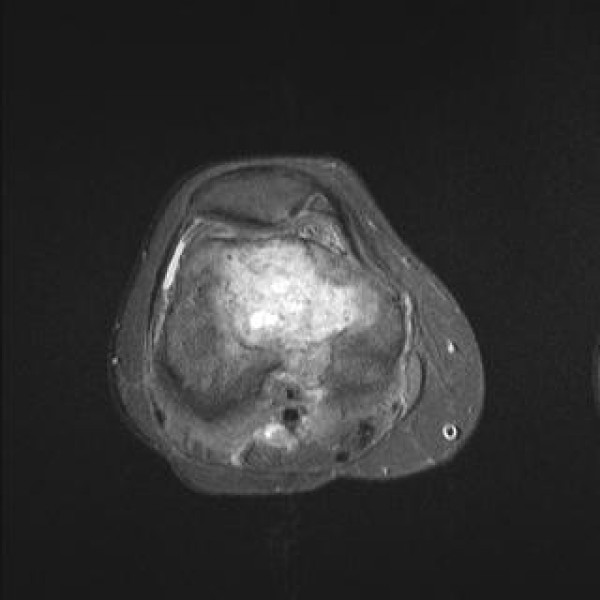
**T1 fat suppression post gadolinium axial view of the distal femur.** The metastasis is bridging the anterior and the posterior femoral cortex and in places is in very close proximity to the popliteal neurovascular structures.

On clinical examination, the right hip was painful with a restricted range of motion and she was unable to weight bear. At the distal part of her right femur a pulsatile mass could be felt but the knee had normal function with no effusion. There was no neurovascular deficit detectable. She scored 8 out of 10 in the visual analogue scale VAS for pain (VAS-P) and 3 out of 10 in the quality of life EuroQolVAS (VAS-EQ) questionnaires[7]. The Harris Hip Score[8] (HHS) was 46, the Beck Depression Index[9] (BDI) 28, the State-Trait Anxiety Index[10] (STAI) was 69, and she was given a score of 3 according to the American Society of Anesthesiologists (ASA)[11]. Radiographs of the pelvis and the entire right femur showed a destructive osteolytic lesion of the femoral head and neck occupying more than 50% of the bony mass with thinning of the medial femoral neck cortex and a large osteolytic lesion in the ipsilateral supracondylar region. Magnetic resonance imaging (MRI) of the right femur revealed a 5.3 cm in diameter bone metastasis within the distal femur which was breaching the posterior cortex and encroaching on the popliteal vessels (Figure 1, Figure 2 Figure 3). A vascular metastatic extrusion in the suprapatellar pouch was also detected (Figure 3). In addition, there was a similar lesion at the level of the lesser trochanter with an ill-defined intra-medullary component and marked cortical reaction (Figure 1). A 2.4 cm mass within the right femoral head extending into the subchondral bone and a second 3.5 cm mass in the femoral neck of lower intensity in STIR coronal views were also detected (Figure 2). MRI of the pelvis revealed vascular metastatic lesions adjacent to the acetabular roof and the posterolateral wall. The rest of the ipsilateral ilium and ischium were intact. Laboratory tests including a full blood count, renal, liver, thyroid function tests, clotting profile and parathyroid hormone levels were all within normal limits.

### Surgical planning and technique

**Figure 4:**
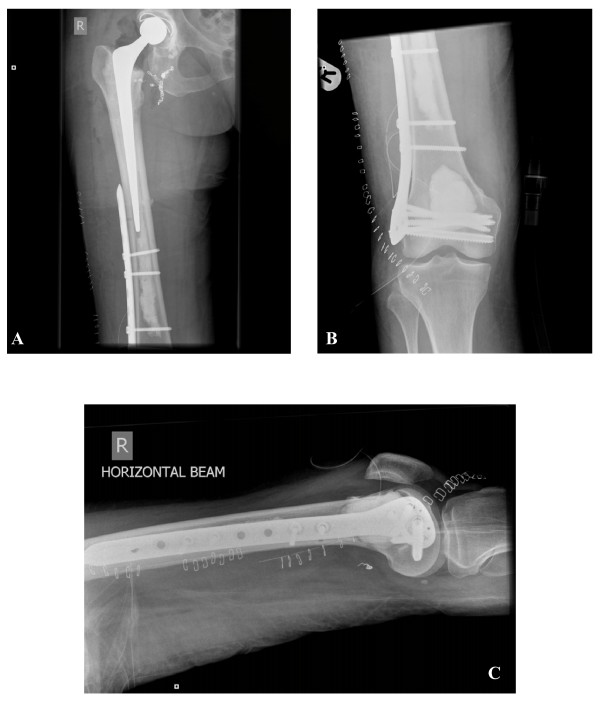
**(a) Anteroposterior view of the proximal femur.** A long cemented Exeter total hip arthroplasty. There is no cement restrictor and the cement has been pressurized to the distal femoral canal. (b&c) Anteroposterior and lateral view of the distal femur. Cementoplasty and locking percutaneous plating of the distal femur.

The two destructive osteolytic lesions of the right femur scored more than 9, according to Mirels classification [12]. An imminent pathological fracture in the proximal and distal femur was thus diagnosed and a multi-disciplinary team meeting took place to discuss the management of this case. The question of long-term survival was raised but no definitive answer was given as the patient had survived this multiple neoplasia syndrome for over 20 years. The dilemma of palliative prophylactic surgery was also debated. The option of femoral nailing (ante- or retrograde) was ruled out due to lack of proximal bony support (femoral neck and acetabular metastasis) and the presence of a distal vascular lesion (exophytic metastasis with vascular MRI signal in the distal femur bridging both the anterior and the posterior distal femoral cortices). Total femoral replacement with a hinged constrained total knee arthroplasty could provide a solution but meticulous anatomical dissection close to the popliteal vessels would be necessary and potentially hazardous. Above knee amputation was an option. However, healing of the stump and loading of the limb would not improve her mobility and quality of life since the metastatic disease was also affecting the ipsilateral hip joint. The distal femur (Mirels classification score 11) was considered to have priority as an imminent fracture was at greater risk compared to the proximal femur (Mirels classification score 9). The patient discontinued interferon therapy for two weeks before surgery. Successful embolisation of the vascular lesions in the acetabulum, proximal and distal femur was performed twenty-four hours prior to surgery. On the operating table the patient was placed in the lateral decubitus position to allow access to both the distal femur and hip joint. A lateral approach to the distal femur was first performed at the level of the lesion and was extended to the femoral condyles in order to allow positioning of a locking plate at that level. The tumour was then approached from the anterior femoral cortex above the patella and resected. Following this, the locking plate was applied percutaneously and was measured in length to overlap proximally the distal end of the cemented femoral prosthesis which was implanted at a second stage. The distal femur was then augmented by methylmethacrylate cement injection to fill the void. No major bleeding was encountered and the cement injection was controlled with continuous radiographic imaging to prevent leakage into the popliteal fossa. Subsequently, the hip joint was opened via a posterior approach. A standard procedure with short rotators and capsular tagging for repair was performed and a cemented Exeter total hip arthroplasty using a long stem (200 mm) was inserted. Meticulous curettage of the metastatic lesions from the acetabular and femoral medullary site was also performed in order to debulk the metastatic mass and replace it with cement. There was no cement restrictor placed and the cement was pressurised to fill the femoral canal as far distally as possible. Venting of the femoral canal was performed through predrilled 4.5 mm holes in the lateral cortex (Figure 4a, 4b, 4c). The patient made an uneventful recovery and started fully weight bearing within a week from surgery. The blood loss (total estimated 700 mls) was within expectations and blood transfusion was not necessary. Within the first 6 weeks the HHS improved to 90, the BDI to 12, the STAI to 48, the VAS-P to 3 and the VAS-EQ to 9. The post operative histopathological examination of the specimens retrieved from the distal femur revealed metastatic renal cell carcinoma. The femoral head and acetabular lesions were also of renal origin whilst the femoral neck lesion was a breast tumour metastasis. The patient continued interferon and bisphosphonate treatment thereafter, and survived for a year having a good quality of life and full mobility.

## Discussion

Our patient presented with a long history of metastatic CS resistant to radio-chemotherapy. We elected to proceed to a combined surgical treatment of both the proximal and distal femoral metastases leaving the unaffected mid femur intact. A percutaneous plate fixation of the distal femur with an intralesional excision and cementoplasty of the metastasis was performed initially. This was combined with a cemented total hip arthroplasty using an Exeter long revision stem and a cementoplasty of the femoral canal. Weber et al[13] recommended curettage with cementation and stabilization for metastatic renal cell carcinomas that are refractory to local adjuvants such as radiotherapy. They showed that this can decrease local progression of the disease. The same authors also advised embolization twelve to thirty six hours pre-operatively for renal cell and thyroid carcinomas especially if the procedure is performed without a tourniquet as in our case. Recently Fuchs et al[14] reported no difference in the long-term survival between wide resection and intralessional curettage of renal cell metastases. However the functional morbidity of a widespread resection and reconstruction and its effect on weight-bearing should be considered and weighed against the potential prolongation of survival. Cemented THA with long stem femoral implant and cemented acetabular component are also recommended for metastatic long bone disease. Uncemented implants are contraindicated as the bone is biologically inert following previous or adjuvant radiotherapy and does not therefore allow ingrowth onto the implants[13]. Cardiopulmonary compromise and thromboembolic events can be minimised by venting the femoral canal and lowering cement pressurisation [15].

In our case, preoperative biopsy or intraoperative frozen section biopsy were deemed unnecessary as the MRI features of the metastatic lesions were vascular and therefore of renal or thyroid origin.

Mirels classification system is a reliable method of predicting the likelihood of a pathological fracture and was used along with the clinical evaluation of the overall functional status of the patient to guide our treatment[16]. We believe that by preserving the femur we have maintained the limb's functional capacity to a significant extent. That would possibly not have been the case if a widespread bone resection or a complex replacement reconstructive procedure had taken place[17]. In addition to, our surgical approach allowed bone cementoplasty enhancement avoiding major resections, excessive blood loss and a high risk of postoperative infection.

## Conclusion

This case reports on CS, a rare disease with multiple skeletal metastases of different origin. The treatment included a combination of cemented arthroplasty, cementoplasty and internal fixation that maintained function and improved our patient's quality of life.

## Consent

Written informed consent was obtained from the patient's husband for publication of this case report and accompanying images in Journal of Medical Case Reports.

## Competing interests

The authors declare that they have no competing interests.

## Authors' contributions

AA collected the clinical details, analysed and interpreted radiographs as well as drafted the first manuscript. EMT prepared the first draft of the manuscript and contributed to the patient's management. IP conducted a literature search and contributed to preparation of the final manuscript. ZG conducted a literature search and contributed to preparation of the final manuscript. JJR contributed to the interpretation of the radiographs and preparation of the manuscript. ET carried out the surgery and helped with preparation of the final manuscript. All authors contributed equally in collecting patient data and editing radiographic images.
